# Pre-implantation genetic diagnosis in an Iranian family with a novel mutation in *MUT* gene

**DOI:** 10.1186/s12881-020-0959-8

**Published:** 2020-02-03

**Authors:** Parham Habibzadeh, Zahra Tabatabaei, Mohammad Ali Farazi Fard, Laila Jamali, Aazam Hafizi, Pooneh Nikuei, Leila Salarian, Mohammad Hossein Nasr Esfahani, Zahra Anvar, Mohammad Ali Faghihi

**Affiliations:** 1 Persian BayanGene Research and Training Center, Shiraz University of Medical Sciences, Shiraz, Iran; 2 Student Research Committee, Shiraz University of Medical Sciences, Shiraz, Iran; 3 Fertility and Infertility Research Center, Hormozgan University of Medical Sciences, Bandar Abbas, Iran; 4 Department of Pediatrics, Shiraz University of Medical Sciences, Shiraz, Iran; 5 Department of Reproductive Biotechnology, Reproductive Biomedicine Research Center, Royan Institute for Biotechnology, ACECR, and Isfahan Fertility and Infertility Center, Isfahan, Iran; 6 Infertility Research Center, Shiraz University of Medical Sciences, Shiraz, Iran; 7 Department of Obstetrics and Gynecology, School of Medicine, Shiraz University of Medical Sciences, Shiraz, Iran

**Keywords:** Organic acidemia, Methylmalonic acidemia, Preimplantation diagnosis, Mutation, missense, Metabolic diseases

## Abstract

**Background:**

Methylmalonic acidemia (MMA), which is an autosomal recessive metabolic disorder, is caused by mutations in methylmalonyl-CoA mutase (*MUT*) gene. As a result, the conversion of methylmalonyl-CoA to succinyl-CoA is impaired in this disorder, leading to a wide range of clinical manifestations varying from no signs or symptoms to severe lethargy and metabolic crisis in newborn infants. Since identification of novel mutations in *MUT* gene can help discover the exact pathogenesis of MMA and also use these disease-causing mutations in prenatal diagnosis, this study was conducted to uncover the possible mutations in an Iranian couple with a deceased offspring clinically diagnosed as having organic acidemia. Moreover, to prevent the occurrence of the mutation in the next pregnancy, we took the advantage of pre-implantation genetic diagnosis (PGD), which resulted in a successful pregnancy.

**Case presentation:**

The affected individual was a 15-month-old boy who passed away due to aspiration pneumonia. The child presented at the age of 3 months with lethargy, protracted vomiting, hypotonia, and decreased level of consciousness. To find the mutated gene, Next Generation Sequencing (NGS) was performed as carrier testing for the parents and the results revealed a novel (private) heterozygous missense mutation in *MUT* gene (c.1055A > G, p.Q352R). After performing PGD on three blastomeres, one was identified as being homozygous wild-type that was followed by successful pregnancy.

**Conclusions:**

Our study identified a novel, deleterious, heterozygous missense mutation in *MUT* gene in a couple and helps to consider the genetic counselling and prenatal diagnosis more seriously for this family with clinical phenotypes of organic acidemia.

## Background

Methylmalonic acidemia (MMA) is an autosomal recessive metabolic disorder [1] that affects 1:48,000 to 1:61,000 newborns in western population [2]. The disease usually presents with lethargy and signs of metabolic crisis including acidosis, hyperketonemia, hypo- or hyperglycaemia and hyperammonemia which may lead to multiorgan failure, coma and even death in the first year of life in affected individuals [3, 4].

In MMA, the conversion of methylmalonyl-CoA to succinyl-CoA is impaired due to pathogenic mutations in methylmalonyl-CoA mutase (*MUT*) gene or genes involved in the synthesis of MUT-cofactor, adenosylcobalamin (AdoCb1), *MMAA*, *MMAB* and *MMADHC* [5–7]. Therefore, elevated levels of methylmalonic acid, a product of fat and protein metabolism, in the blood, urine and cerebrospinal fluid (CSF) contribute to the life-threating symptoms seen in MMA [8, 9].

Mutations in *MUT* gene, encoding methylmalonyl-CoA mutase, account for 60–70% of MMA cases. Mutations referred as mut^0^ are associated with completely abolished enzyme activity and mut^−^ indicates some residual enzyme activity [10].

Several specific mutations have been reported among various ethnic groups. For example, p.E117X, p.L494X, p.R93H, p.R369H, p.G648D, I739T, p.R727X, and c.385 + 5G > A were identified in Japanese patients [8, 11, 12], and p.L140P, p.A141T, p.G161 V, p.W309G, p.I505T, p.Q514K, p.I597R, and p.G723D in Chinese patients [13]. Kumari, et al, identified 23 novel mutations within exons 2, 9, 11, and 12 of *MUT* gene among Indian patients [6]. Among Saudi patients, p.Y110C and p.Q734X were found [14]. Ahmadloo, et al, reported a novel variation in the *MUT* intron 12 (c.2125-3C > G) among Iranian cases with MMA [15]. In addition, in a recent study, Shafaat, et al, found five novel pathogenic mutations in *MUT* gene (c.805delG, c.693delC, c.223A > T, c.668A > G, and c.976A > G) [16]. However, there are limited studies on Iranian patients and molecular genetic approaches in this population could play pivotal role in identification of novel pathogenic variants and genetic counselling.

Pre-implantation genetic diagnosis (PGD) is a non-invasive approach to prenatal diagnosis in couples with a genetic disorder. The aim is to increase the probability of having a healthy offspring. The technique could be applied when a certain genetic mutation or a structural chromosomal abnormality is found and confirmed in the parents. The couple will undergo in vitro fertilization (IVF) followed by genetic analysis on produced embryos to select the unaffected one [17, 18].

In the present study, we report on a novel mutation in the *MUT* gene in a couple heterozygous for this mutation along with the clinical and laboratory findings of a deceased offspring in the family clinically diagnosed as having organic academia.

## Case presentation

The affected individual was a 15-month-old boy who passed away due to aspiration pneumonia. He was born to consanguineous parents, who were first-degree cousins. The child presented at the age of 3 months with lethargy, protracted vomiting, hypotonia, and decreased level of consciousness and was admitted to the pediatric intensive care unit (PICU). Arterial blood gas analysis revealed a pH of 7.02 (normal range: 7.35–7.45), pCO_2_ of 17.6 (normal range: 35–45) mm Hg and [HCO_3_] of 5.3 (normal range: 20–28) mmol/L. The acute attack had been managed with hydration and administration of IV bicarbonate in order to correct the acidosis. Other routine laboratory investigations revealed no abnormalities with a hemoglobin of 11.3 (normal range for 3–6-month-old infants: 9.5–14.1) g/dL, total white blood cell count of 6700/mm^3^ (normal range for 3–6-month-old infant: 6000–17,500/mm^3^), blood urea nitrogen of 15 (normal range: 5–20) mg/dL, creatinine of 0.5 (normal range: 0.2–0.5) mg/dL, sodium of 135 (normal range: 135–145) mEq/L, potassium of 4.6 (normal range: 3.5–5) mEq/L, and blood sugar of 92 (normal range: 65–99) mg/dL. Liver function enzyme levels were also within normal range. Analysis of the CSF revealed no abnormalities. Evaluation for metabolic disease using tandem mass spectrometry revealed elevated levels of propionylcarnitine (10.3 μmol/L, normal range < 2.5 μmol/L) and increased propionylcarnitine/acylcarnitine ratio (0.53, normal range < 0.13). Methylmalonic acid level was not determined. The patient was subsequently discharged with L-carnitine, vitamin B_12_ and chronic alkaline therapy.

In subsequent evaluations the patient had an improvement in the overall condition. However, growth parameters were not within normal range. Weight being 6 kg and length being 66 cm were both lower than one percentile for age. The patient also failed to reach developmental milestones. At the age of 9 months, he had head lag and was not able to sit alone, could not transfer objects hand to hand and did not say two-syllable words. Furthermore, he had two other episodes of acute attacks, which had led to hospital admission. At the age of 15 months, he presented with tachypnea, fever, cough, and decreased level of consciousness. Chest x-ray was in favor of aspiration pneumonia. Intravenous antibiotics were administered and alkaline therapy was started. However, the patient showed no clinical response to the treatment.

To determine the causative genetic mutation in the family, an unbiased next generation DNA sequencing which covered the entire coding exons (whole exome sequencing, WES) was carried out. Peripheral blood was obtained from parents and was used to isolate genomic DNA. WES was done utilizing next generation sequencing on an Illumina NextSeq500 (Illumina, USA). The results were subsequently analyzed with BWA aligner [19], GATK [20] and ANNOVAR [21]. To identify common probably pathogenic mutations in the couple, carrier screening was performed on annotated data. Filtering was accomplished based on local (sample size: 1183 unrelated individuals) and other available population databases (such as ExAC browser, gnomAD and Kaviar VARiants).

WES was performed with a mean target coverage of 100×. Results revealed a novel heterozygous missense mutation in *MUT* gene (c.1055A > G, p.Q352R) in both male and female partners. Homozygous mutations in this gene are compatible with MMA and since MMA has an autosomal-recessive pattern of inheritance, this finding suggests that the child had probably had MMA by inheriting both mutated alleles.

To confirm the presence and the pattern of inheritance of the novel identified mutation, peripheral blood samples from the couple and their parents and siblings were taken; DNA was extracted and used for Sanger sequencing. QIAamp DNA Blood Mini kit (Cat No./ID: 51104, Germany) was used for genomic DNA extraction. The following primers were used to amplify exon five of *MUT* gene as well as its flanking intronic sequences to look for the mutation—Forward: 5′ TCAGCACTACAGGGAAGCTAG 3′ and Reverse: 5′ GACCTAACGTTACTATTTTAGGTTGT 3′.

**Fig. 1:**
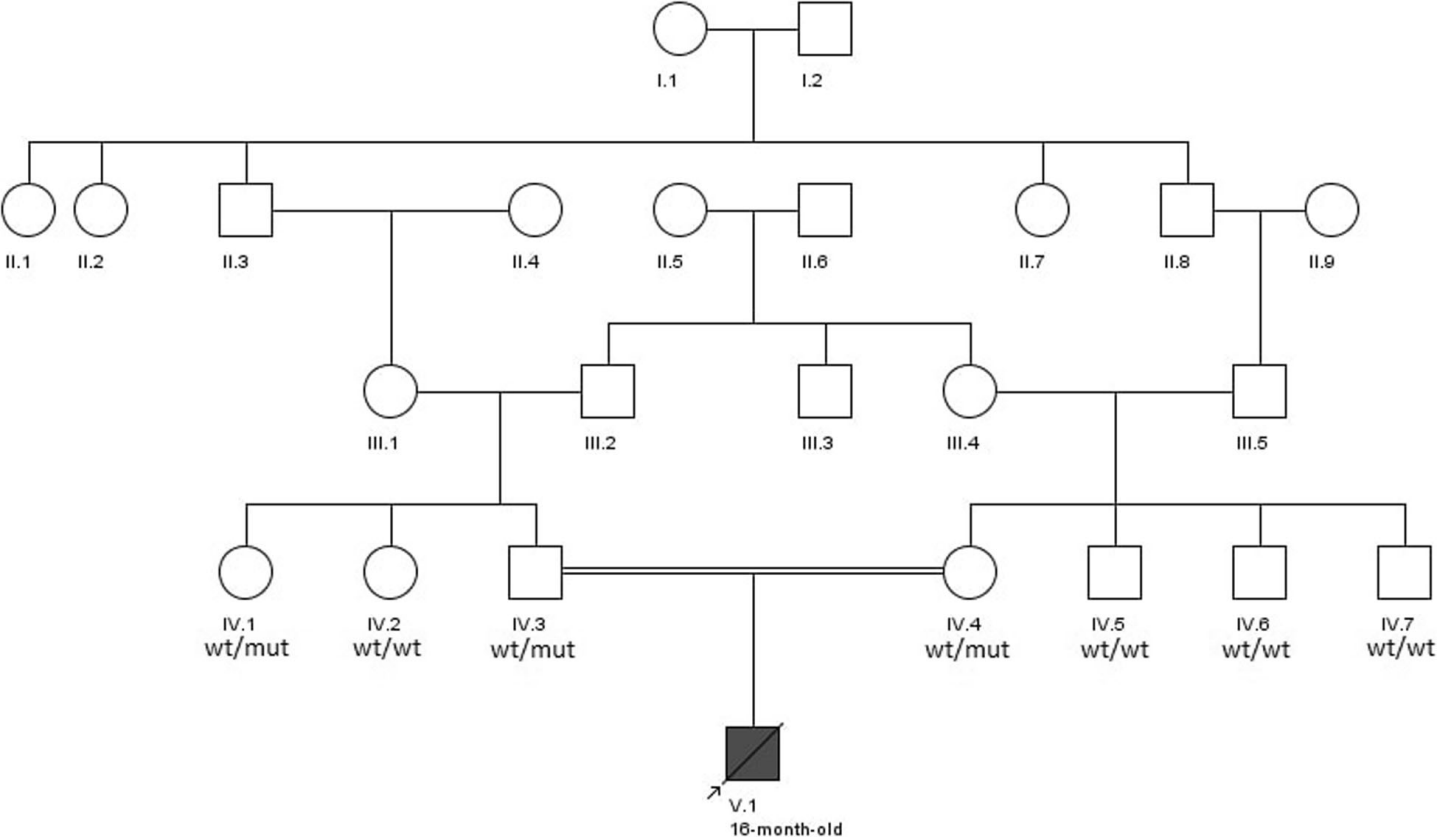
The family pedigree depicting the mutation in *MUT* gene. ‘wt’ and ‘mut’ represent the wild type and pathogenic alleles, respectively

**Fig. 2:**
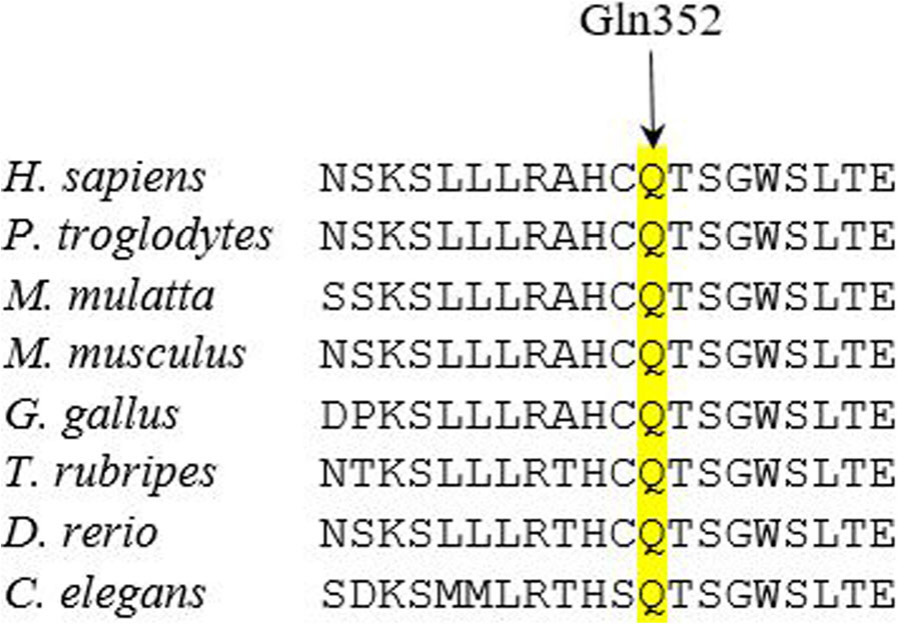
Comparative amino acid alignment of *MUT* protein across different kingdoms. The highly conserved glutamine residue is highlighted

The amplified DNA was sequenced from both directions using Sanger Sequencing kit (ABI BigDye Terminator Cycle Sequencing Kit, Applied Biosystems®, USA) according to the company protocol. Sanger sequencing confirmed the presence of mutation in heterozygous form in both parents. The parents’ siblings were either heterozygous carriers or homozygous for the wild type allele (Fig. 1). This mutation is a variant of uncertain significance and has not previously been reported. However, bioinformatics software programs such as Polyphen, SIFT, LRT, Mutation Taster, FATHMM, Radial SVM, and Mutation Assessor software have predicted that this variant will be damaging. Moreover, comparative amino acids alignment using multiple sequence alignment revealed that this amino acid is highly conserved among different species (Fig. 2).

Ovarian stimulation was performed using the antagonist protocol. The couple underwent intra-cytoplasmic sperm insemination. Among four retrieved oocytes, three were fertilized and produced three 8-cells embryos on day three. One blastomere from each cleavage stage embryo was isolated and transferred into a sterile 0.2-mL PCR tube containing 5 μL of PBS and kept at − 80 °C in this condition until testing. DNA amplification across the exon 5 of the *MUT* gene of each of the blastomeres was done using whole-genome amplification (WGA) reaction.

Whole-genome amplification reaction was done by REPLI-g Single Cell kit (QIAGEN). Briefly, samples were mixed with 3 μL of denaturation buffer and incubated at 65 °C for 10 min, followed by inactivation by adding 3 μL of stop solution. Then, master mix containing REPLI-g sc DNA polymerase was added to the denatured DNAs. Finally, the mixture was incubated at 30 °C for 8 h followed by heat inactivation at 65 °C for 3 min. The products were kept at 4 °C until PCR amplification. DNA amplification of one of the embryos failed. DNA amplification was successful with the other two blastomeres. There was no amplification in the medium blank controls ran simultaneously.

**Fig. 3:**
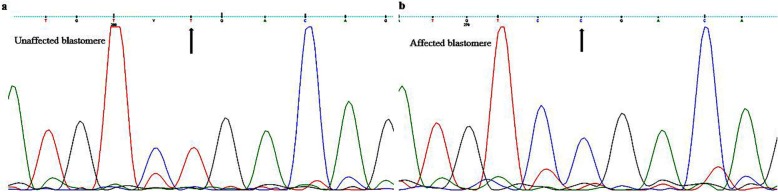
Sequence chromatograms of (**a**) unaffected and (**b**) affected blastomeres

PCR was performed on the products for the amplification of the exon 5 of the *MUT* gene. Sanger sequencing was then performed on the PCR products to determine the *MUT* genotype of the embryos using 3130xl Genetic Analyzer (Applied Biosystems). Among the two successfully amplified blastomeres, one was diagnosed as homozygous for the wild-type allele and the other was homozygous for the mutated allele (Fig. 3**)**. The unaffected embryo was then transferred. Single embryo transfer (SET) resulted in a successful implantation and clinical pregnancy confirmed by detection of gestational sac and fetal heart at 4–5 weeks after embryo transfer by transvaginal ultrasonography. The embryo was confirmed as unaffected (homozygous wild type) after prenatal diagnosis done by amniocentesis at 15 weeks of gestation.

## Discussion and conclusion

In the first decade of 2000 the MMA mortality rate was around 40% [6, 22], which indicates that the disease has a poor prognosis and that without treatment the condition can lead to long-term neurodevelopmental impairments. Therefore, early detection and therapy may effectively help the patients. Liver transplantation has been proposed as a therapeutic modality. However, some discrepancies on the feasibility of liver transplantation exist in the literature [2, 4].

Autosomal-recessive disorders, such as MMA, are more prevalent in countries with high rate of consanguineous marriages. The rate of consanguineous marriage in Iran exceeds 35% [16, 23]. In a 10-year study conducted in Iran on MMA patients, 80% of cases were offspring of consanguineous marriages [24]. These observations highlight the pivotal role of genetic analysis to find and report the novel pathogenic variants for prevention of the disease transmission in suspected families and establish disease control policies, such as newborn screening in such societies.

Identification of novel disease-causing mutations in the past decade is largely attributed to the advances made in next generation sequencing technologies, so that 361 deleterious mutations have so far been identified and reported in *MUT* gene (*http://www.hgmd.cf.ac.uk*). Most of these mutations are missense mutations and confer different effects on stability and activity of the protein [25].

Herein, we studied a couple with an offspring who died of the sequelae of organic acidemia. On account of the fact that methylmalonic acid level was not determined, both MMA and propionic academia (PA) could be considered in this patient. Therefore, since PA can be caused by large deletions in *PCCA* and *PCCB* genes, which could be missed by WES, PA cannot be completely discarded in this patient [2]. However, using WES, we identified a deleterious missense mutation in *MUT* gene in a highly conserved residue changing the 352th amino acid from glutamine to arginine. Unfortunately, we did not have access to any samples from the deceased individual. However, the identification of the mutation in heterozygous form in both parents suggested the presence of a homozygous mutation in the offspring who had presented with the symptoms compatible with organic acidemia. Nonetheless, it should be acknowledged that the identified variant might be a marker rather than the cause of the disease. Once the mutation was confirmed in the couple, based on parents’ request, insisting on proceeding with the identified variant of uncertain significance, we took the advantage of PGD (following IVF/ICSI) to prevent the transfer of the deleterious mutation to the embryo to be implanted. Between the two examined embryos, one was unaffected and thus transferred to the uterus. Clinical pregnancy was achieved and amniocentesis confirmed homozygous wild-type *MUT* allele in the fetus. In this way, the strategy used effectively prevented the transmission of deleterious allele to the next generation. In a similar way, the strategy can be used efficiently in preventing other similar genetic disorders.

## Data Availability

All data are available from the corresponding author on request.

## Abbreviations

CSF: Cerebrospinal fluid
IVF: In vitro fertilization
MMA: Methylmalonic acidemia
MUT: Methylmalonyl-CoA mutase
NGS: Next Generation Sequencing
PA: Propionic academia
PGD: Pre-implantation genetic diagnosis
PICU: Pediatric intensive care unit
SET: Single embryo transfer
WES: Whole exome sequencing
WGA: Whole-genome amplification
